# Supplementary material for: Determination and Risk Assessment of Flavor Components in Flavored Milk
Source: Foods. 2023 May 26;12(11):2151. doi: 10.3390/foods12112151 (PMC10252675; doi:10.3390/foods12112151)
Supplement: Supplementary file 1 [file foods-12-02151-s001.zip › Table S1 Residents flavored milk consumption questionnaire.pdf]

Residents flavored milk consumption questionnaire

Q1. What's your sex?

☐ Male

☐ Female

Q2. What's your age?

☐< 10 ☐11~18 ☐18~24 ☐25~30 ☐31~40 ☐41~50 ☐51~60 ☐>61

Q3. What is your weight (kg)? ____________

Q4. What is your current occupation?

☐ School students

☐ Government/government cadres/civil servants

☐ Ordinary staff (office/office workers)

☐ Professionals (such as doctors/lawyers/stylistics/journalists/teachers)

☐ Ordinary workers (such as factory workers/manual workers)

☐ Business service workers (such as salespeople/shop staff/waiters,)

☐ Freelancer

☐ Others

Q5. What is your address?______________________

Q6. What kind of flavored milk do you usually drink? (Multiple choices)

☐Strawberry flavor ☐Chocolate flavor ☐Peanut flavor ☐Juice flavor

☐Wheat flavor ☐ Mango flavor ☐Cream flavor ☐Orange flavor

☐Pineapple flavor ☐Mocha flavor ☐Peach flavor ☐Black sesame flavor

☐Banana flavor ☐Walnut flavor ☐Red date flavor ☐Red bean flavor

☐Milk flavor ☐Yellow peach flavor ☐Hamimelon flavor ☐Blueberry flavor

☐Raspberry flavor ☐Passion fruit flavor ☐Apple flavor ☐Coconut flavor

☐Others

Q7. How many times you drink flavored milk on average a month? ___________

Q8. What is the capacity or weight of flavored milk you drink each time? (For example : 250ml, 300g)_______________
