# Supplementary material for: Determination and Risk Assessment of Flavor Components in Flavored Milk
Source: Foods. 2023 May 26;12(11):2151. doi: 10.3390/foods12112151 (PMC10252675; doi:10.3390/foods12112151)
Supplement: Supplementary file 1 [file foods-12-02151-s001.zip › Table S2 Sample characteristics of flavored milk consumption questionnaire.pdf]

Table S2. Sample characteristics of flavored milk consumption questionnaire

Variable	Category	Quantity	Frequency
Gender	male	839	39.80%
	female	1269	60.20%
Age	<18	55	2.61%
	18~24	1032	48.96%
	25~30	533	25.28%
	31~40	305	14.47%
	41~50	126	5.98%
	>51	57	2.70%
Occupation	Ordinary workers	64	3.04%
	Ordinary staff	265	12.57%
	Others	38	1.80%
	Business service workers	42	1.99%
	School students	1210	57.40%
	Government/government cadres	111	5.27%
	Professionals	271	12.86%
	Freelancer	107	5.08%
Area	East China	349	16.56%
	South China	135	6.40%
	Central China	162	7.69%
	North China	858	40.70%
	Northwest	220	10.44%
	Southwest	283	13.43%
	Northeast	101	4.79%
Flavor	Strawberry flavor	775	10.90%
	Milk flavor	694	9.76%
	Yellow peach flavor	507	7.13%
	Chocolate flavor	504	7.09%

	Red date flavor	468	6.58%
	Mango flavor	442	6.22%
	Wheat flavor	381	5.36%
	Other flavors	63~316	0.89%~4.44%
