# Supplementary material for: Determination and Risk Assessment of Flavor Components in Flavored Milk
Source: Foods. 2023 May 26;12(11):2151. doi: 10.3390/foods12112151 (PMC10252675; doi:10.3390/foods12112151)
Supplement: Supplementary file 1 [file foods-12-02151-s001.zip › Table S4 Linear equation, R2 and detection limit of flavor concerned components.pdf]

Table S4 Linear equation, R2 and detection limit of flavor concerned components

Compounds	Linear equations (μg L^{-1})	R^2	LOD	LOQ
2-Methylpropanal	y = 3837.9x + 116107	0.9960	0.006	0.021
Ethyl 3-methylbutyrate	y = 1073846.59 x - 1194922.16	0.9981	0.008	0.027
1-Hexanol	y = 197823x - 846541	0.9984	0.057	0.190
Hexanoic acid, 2-propenyl ester	y = 565723.87 x - 7832214.43	0.9967	0.017	0.056
2,3,5-Trimethylpyrazine	y = 889315.90x - 12121223.06	0.9975	1.667	5.556
Furfural	y = 770367.78 x - 8779555.11	0.9976	0.012	0.040
benzaldehyde	y = 2520993.18 x-35511855.34	0.9956	0.007	0.025
Linalool	y = 696561.43 x - 7732584.50	0.999	0.014	0.048
5-Methylfurfural	y = 587680.93 x-7276403.78	0.9976	0.010	0.032
Benzyl acetate	y = 975571.14 x-9601990.44	0.9986	0.005	0.016
Methyl salicylate	y = 804217.84 x - 9249485.76	0.9979	0.007	0.024
benzenemethanol	y = 67584.93 x - 1314265.10	0.9921	0.048	0.160
Maltol	y = 38450x - 5820.8	0.9922	0.158	0.527
Methyleugenol	y = 392653.84 x - 9425563.72	0.9903	0.008	0.026
Phenol,2-methoxy-4-(2-propenyl)-	y = 215643.96 x - 5105057.48	0.9904	0.020	0.068
