# Supplementary material for: Determination and Risk Assessment of Flavor Components in Flavored Milk
Source: Foods. 2023 May 26;12(11):2151. doi: 10.3390/foods12112151 (PMC10252675; doi:10.3390/foods12112151)
Supplement: Supplementary file 1 [file foods-12-02151-s001.zip › Table S5 Recovery and precision of 15 concerned components.pdf]

Table S5 Recovery and precision of 15 concerned components

Compound	Add scalar (μg L^{-1})			Recovery (%)				RSD（%）			
	Level1	Level2	Level3	Level1	Level2	Level3	Average recovery	Level 1	Level 2	Level 3	Average RSD
2-Methylpropanal	136.86	522.44	1968.62	136.86	104.49	98.43	113.26	2.74	4.85	3.01	3.53
Ethyl 3-methylbutyrate	11.22	18.76	100.46	112.20	93.82	100.46	102.16	2.96	2.75	3.43	3.05
1-Hexanol	21.21	37.17	455.43	106.04	74.33	91.09	90.49	3.13	1.65	2.51	2.43
Hexanoic acid, 2-propenyl ester	29.89	47.92	558.79	149.44	95.85	111.76	119.01	1.39	3.86	5.37	3.54
2,3,5-Trimethylpyrazine	28.55	48.48	578.91	142.74	96.96	115.78	118.49	1.65	1.65	2.02	1.77
Furfural	24.69	44.85	541.50	123.46	89.70	108.30	107.15	1.69	1.54	2.95	2.06
Benzaldehyde	28.87	50.75	594.04	144.33	101.5	118.81	121.54	1.69	3.38	1.33	2.13
Linalool	26.22	47.38	545.28	131.11	94.77	109.06	111.65	1.35	1.59	2.98	1.97
5-Methylfurfural	27.13	48.03	588.36	135.67	96.06	117.67	116.47	1.90	1.63	3.71	2.41
Benzyl acetate	24.10	46.04	575.75	120.49	92.06	115.15	109.24	2.00	2.80	1.05	1.95
Methyl salicylate	22.86	45.69	688.16	114.30	91.39	133.63	113.11	1.02	1.65	3.23	1.96
Benzenemethanol	31.29	45.63	561.44	156.45	91.27	112.29	120.00	4.80	2.87	2.58	3.42
Maltol	88.48	195.58	533.28	88.48	97.79	106.66	97.64	1.72	9.83	5.75	5.77
Methyleugenol	33.70	43.82	611.63	168.49	87.65	122.33	126.16	3.06	0.98	8.81	4.28
Phenol,2-methoxy-4-(2-propenyl)-	33.75	43.54	595.33	168.77	87.08	119.07	124.97	1.88	1.49	11.31	4.89
